# The Role of Osteopontin in Microglia Biology: Current Concepts and Future Perspectives

**DOI:** 10.3390/biomedicines10040840

**Published:** 2022-04-03

**Authors:** Dennis-Dominik Rosmus, Clemens Lange, Franziska Ludwig, Bahareh Ajami, Peter Wieghofer

**Affiliations:** 1 Institute of Anatomy, Leipzig University, 04103 Leipzig, Germany; dennis-dominik.rosmus@medizin.uni-leipzig.de; 2 Eye Center, Freiburg Medical Center, University of Freiburg, 79106 Freiburg, Germany; clemens.lange@augen-franziskus.de (C.L.); franziska.ludwig@uniklinik-freiburg.de (F.L.); 3 Ophtha-Lab, Department of Ophthalmology, St. Franziskus Hospital, 48145 Muenster, Germany; 4 Department of Microbiology and Immunology, Oregon Health and Science University, Portland, OR 97239, USA; ajami@ohsu.edu; 5 Cellular Neuroanatomy, Institute of Theoretical Medicine, Medical Faculty, Augsburg University, 86159 Augsburg, Germany

**Keywords:** microglia, macrophages, osteopontin, SPP1, inflammation, neurodegeneration, multiple sclerosis, Alzheimer’s disease, AMD, retina, CNS

## Abstract

The innate immune landscape of the central nervous system (CNS), including the brain and the retina, consists of different myeloid cell populations with distinct tasks to fulfill. Whereas the CNS borders harbor extraparenchymal CNS-associated macrophages whose main duty is to build up a defense against invading pathogens and other damaging factors from the periphery, the resident immune cells of the CNS parenchyma and the retina, microglia, are highly dynamic cells with a plethora of functions during homeostasis and disease. Therefore, microglia are constantly sensing their environment and closely interacting with surrounding cells, which is in part mediated by soluble factors. One of these factors is Osteopontin (OPN), a multifunctional protein that is produced by different cell types in the CNS, including microglia, and is upregulated in neurodegenerative and neuroinflammatory conditions. In this review, we discuss the current literature about the interaction between microglia and OPN in homeostasis and several disease entities, including multiple sclerosis (MS), Alzheimer’s and cerebrovascular diseases (AD, CVD), amyotrophic lateral sclerosis (ALS), age-related macular degeneration (AMD) and diabetic retinopathy (DR), in the context of the molecular pathways involved in OPN signaling shaping the function of microglia. As nearly all CNS diseases are characterized by pathological alterations in microglial cells, accompanied by the disturbance of the homeostatic microglia phenotype, the emergence of disease-associated microglia (DAM) states and their interplay with factors shaping the DAM-signature, such as OPN, is of great interest for therapeutical interventions in the future.

## 1. Introduction

Complex systems such as the body of mammals are comprised of different organs and tissues whose proper function is orchestrated and surveilled by a central nervous system (CNS) acting as the “supervisor”. Despite its relevance, the CNS exhibits a high vulnerability towards damage, which may be caused by extrinsic factors or intrinsic dysfunction leading to irreversible loss of function. One of the systems that is intended to protect the CNS is its innate immune system consisting of different myeloid cell types [1,2]. Due to the compartmentalization of the CNS and its adjacent tissues, microglia, the tissue-resident macrophages of the CNS parenchyma may be distinguished from macrophages populating the CNS boundaries, such as the meninges, the perivascular space and the choroid plexus [3,4,5]. Moreover, the retina, which develops from an evagination of the diencephalon [6], and the other compartments of the eye harbor distinct macrophage populations with important functions during homeostasis and disease [7,8,9]. In contrast to extraparenchymal CNS-associated and ocular macrophages, that are located at potential entry sites to the CNS and represent the first line of defense against invading pathogens, microglia are evenly distributed across the nervous tissue surveilling their local microenvironment [10,11]. Beside their classical immune functions, research in recent years has extended our horizon on points of a deeper understanding of their non-immune functions [12]. To ensure CNS maintenance, microglia closely interact with other cell types, including glial cells and neurons. These cell–cell interactions are mediated by receptor–ligand interactions on the one side and soluble factors on the other side.

One of these factors is the matricellular protein Osteopontin (OPN), which serves as a “bridge builder” in the CNS by interacting with different types of receptors and proteins of the extracellular matrix and is broadly expressed in nearly all CNS cell types [13]. Research in the recent years has found OPN to be upregulated in several neuroinflammatory and neurodegenerative disorders, including multiple sclerosis (MS) [14], Alzheimer’s disease (AD) [15] and age-related macular degeneration (AMD) [16,17,18]. Given the fact that the expression of OPN is strongly conserved across several CNS pathologies, research efforts have been undertaken to investigate the potential of OPN to serve as a therapeutic target [19]. This may be of particular interest for microglia research, as OPN is known to exert several effects on microglial polarization and function [20,21]. This review summarizes our current knowledge about the interplay between OPN and microglia in the CNS under homeostatic and pathological conditions with a particular emphasis on the signaling pathways by which OPN influences microglia states and functions.

## 2. Microglia in the CNS–Old Dogs with New Tricks

**Figure 1:**
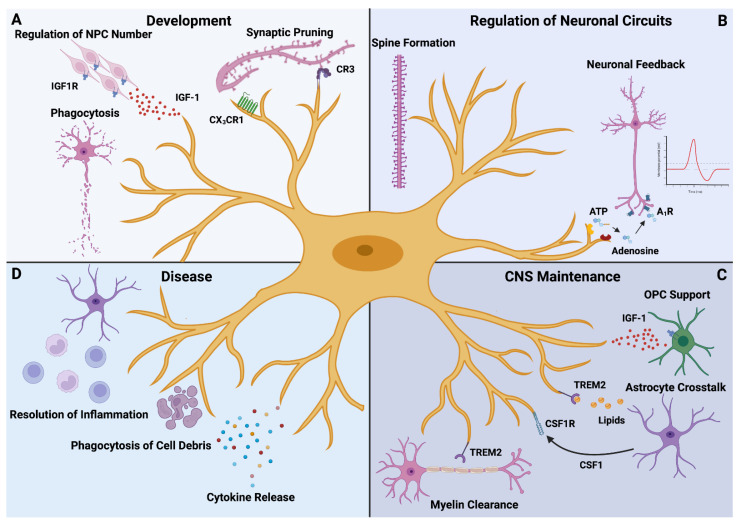
Functions of Microglia in Health and Disease. (**A**) During prenatal development, microglia essentially contribute to the regulation of neuronal cell numbers by removing redundant neurons through phagocytosis and regulating neuronal progenitor cell (NPC) proliferation. Moreover, they participate in the process of synaptic pruning using a plethora of receptors such as CX_3_CR1 or complement receptor 3 (CR3). (**B**) Postnatally, microglia are in intimate contact with neurons and thereby promote spine formation and form ATP-dependent negative feedback loops to tightly regulate neuronal activity. (**C**) Microglia interact with other non-neuronal cell types. They support oligodendrocyte progenitor cells (OPCs) by secreting insulin-like growth factor 1 (IGF-1) and interact with astrocytes through the CSF1–CSF1R axis and the TREM2-dependent uptake of cholesterol-containing lipids. (**D**) Under pathological conditions, microglia are key players as they are capable of secreting disease-modulating cytokines and phagocytizing cell debris. Finally, they orchestrate the resolution of inflammatory responses in the CNS through the interaction with several immune and non-immune cells.

Since their first description in the first half of the 20th century by the Spanish neuroscientist Pío del Río-Hortega [22,23], our understanding of the CNS resident immune cells has undergone an enormous evolution, fueled by breakthrough discoveries in the last 20 years (Figure 1) [24]. Today, microglia are regarded as the tissue-resident macrophages of the CNS parenchyma. They originate from early erythromyeloid precursor cells in the yolk sac, develop in a PU.1- and Interferon Regulatory Factor 8 (IRF8)-dependent manner [25,26] and enter the developing murine CNS as early as at embryonic day 9.5 (E9.5) [25]. During prenatal development, microglia sequentially acquire their adult phenotype through stepwise changes in gene expression and regulatory networks [27,28], which is related to their different tasks at different developmental stages (Figure 1A). One of these tasks is the regulation of neuron cell numbers in the developing CNS [29] and the removal of redundant neurons undergoing apoptosis by the means of phagocytosis, as shown in the zebrafish [30]. Moreover, the refinement of emerging neural circuits largely relies on microglia as these cells can eliminate redundant synapses [31]. This important function involves several mechanisms, including complement factor 3 (C3)-mediated pruning in the retina [32] and purinoreceptor-dependent synaptic plasticity in the visual cortex [33]. In its sum, these processes lead to the maturation of CNS neural networks and thereby contribute to the functionality of the healthy CNS.

In the adult CNS, microglia display a high grade of multifunctionality as these cells are engaged in a variety of physiological processes (Figure 1B). Through their highly motile processes, microglia are constantly sensing their microenvironment [10,11] and contacting neuronal synapses [34,35,36]. Therefore, microglia essentially contribute to the postnatal maturation of neural circuits [37] and hence emerge as regulators of neuronal activity. These findings are complemented by a recent study revealing a negative feedback pathway by which microglia suppress overwhelming neuronal activity through an adenosine receptor A_1_R-dependent mechanism [38]. Besides these interactions with neuronal cells, microglia communicate with other cell types in the CNS to accomplish their goals (Figure 1C). In the process of myelination, microglia provide support to oligodendrocytes and their precursors by secreting insulin-like growth factor (IGF-1) [39]. On the other hand, microglia stand in steady contact with astrocytes, which is mediated by a plethora of molecules, e.g., colony-stimulating factor 1 (CSF1) [12]. Moreover, astrocytes supply microglia with cholesterol that is essential for the maintenance of the homeostatic state and relies on interaction with lipoparticles dependent on Triggering Receptor Expressed on Myeloid cells (TREM) 2 [12]. Finally, microglia can remove myelin debris in the white matter of aged mice which is accompanied by a unique gene expression signature [40]. Collectively, the recent discoveries paint a complex picture of microglia as crucial players for an adequate CNS function.

However, this picture may dramatically change when the homeostatic state is disturbed by pathological insults (Figure 1D). Here, microglia were shown to play important roles during CNS pathology ranging from the secretion of disease-modulating cytokines and the removal of apoptotic neurons to the orchestration of inflammatory responses [24,41]. Under neuroinflammatory and neurodegenerative conditions, the microglia compartment of the CNS is characterized by the emergence of disease-associated microglia (DAMs) with unique gene expression signatures [7,8,28,42,43]. Given the importance of microglia dysfunction in the pathophysiology of CNS disease, mapping distinct microglia states to their physiological function under healthy and diseased conditions may represent a useful strategy to identify therapy targets [24,44]. Furthermore, recent discoveries suggest that the dysregulation of intercellular signaling pathways in microglia under pathological conditions may serve as a driving force behind the development of potentially dysfunctional microglial states even worsening CNS integrity [12,45]. Therefore, identifying these crucial factors and untangling the exact molecular pathways by which they cause the shift from homeostatic microglia to DAMs will not only provide deeper insights into microglia biology, but also render new efforts possible to develop microglia-based therapeutic approaches [44]. One of these potential factors is Osteopontin whose role in the CNS will be discussed subsequently.

## 3. The Role of Osteopontin in the Central Nervous System

Osteopontin is a multifunctional protein which is broadly expressed across several tissues, including the CNS [21]. The high grade of its multifunctionality is also reflected by its name history. OPN was originally described as a sialoprotein in the matrix of bones leading to the prefix “osteo-” meaning bone in Greek [19,46]. On the other hand, the suffix *“*-pontin*”* (originating from the Latin word “pons” meaning bridge) already indicates its capability of forming “bridges” across cells and molecules [19]. The multifunctionality of OPN is further highlighted by the fact that OPN was discovered in several tissues with different functions explaining the variety of different names for this molecule, including early T cell activation (ETA1) gene [47], bone sialoprotein 1 (BSP-1), OPN and, more formally, secreted phosphoprotein 1 (SPP1) [19]. To fulfill this important task, OPN exhibits a sophisticated molecular structure. It was the study of Cantor and colleagues [47] that already highlighted the importance of the arginine–glycine–aspartic acid tripeptide sequence (RGD motif) for the interaction of OPN with other cells, including macrophages. This RGD motif serves as the binding site for several $\alpha_{V}$ integrin receptors [48,49]. Moreover, the OPN molecule contains a cryptic SVVYGLR-domain which is mandatory for the interaction with $\alpha_{4}\beta_{1}$, $\alpha_{9}\beta_{1}$ and $\alpha_{4}\beta_{7}$ integrins [50]. Notably, these integrins bind to the SVVYGLR-domain after thrombin-mediated cleavage [50]. In general, the thrombin cleavage generates two OPN fragments named N-terminal and C-terminal OPN [21]. Whereas the N-terminal fragment mainly interacts with integrins, the C-terminal fragment is considered to interact with the hyaluronic acid receptor cluster of differentiation 44 (CD44) in an RGD independent manner [49,51]. Interestingly, OPN may also undergo posttranslational processing that is mediated by matrix metalloproteases (MMPs), including MMP3, MMP7 and MMP12 [52,53] that leads to OPN fragments with different immunomodulatory effects in comparison to full length OPN [53]. Additionally, several studies have identified an intracellular variant of OPN that is expressed in some immune cell populations including dendritic cells (DCs) [54,55].

In recent years, OPN has raised the attention of different disciplines in biomedical research as this protein has been found to be upregulated in several autoimmune inflammatory diseases, ranging from rheumatological diseases such as systemic lupus erythematosus [56] to chronic inflammatory bowel disease such as Crohn’s disease [57]. Moreover, OPN has been linked to tumor progression in solid malignancies [58]. In the CNS, OPN was originally found to be upregulated in lesions of patients suffering from multiple sclerosis (MS), a devastating autoimmune disease causing neurological deficits that may ultimately culminate in invalidity or death [14]. Furthermore, OPN-deficient mice with experimental autoimmune encephalomyelitis (EAE), a common mouse model for MS, have been found to exhibit a less severe phenotype [14] with a higher percentage of remissions when compared to EAE wildtype mice as well as to be protected from EAE-related death [14]. Mechanistically, OPN increases T cell survival in EAE through the inhibition of transcription factor forkhead box O3A (Foxo3a), the concomitant activation of the nuclear factor kappa-light-chain-enhancer of activated B cells (NF-κB) signaling pathway and the altered expression of anti-apoptotic proteins [59]. Despite these novel insights into the molecular mechanisms driving the effects of OPN on adaptive immune cells in EAE, the exact role of innate immune cells such as microglia and the changes that are mediated by OPN are still far less understood. Therefore, as a next step, we will focus on the known effects of OPN on microglia and the molecular pathways by which these effects are mediated.

## 4. Osteopontin and Microglia—An Intimate Interplay

**Figure 2:**
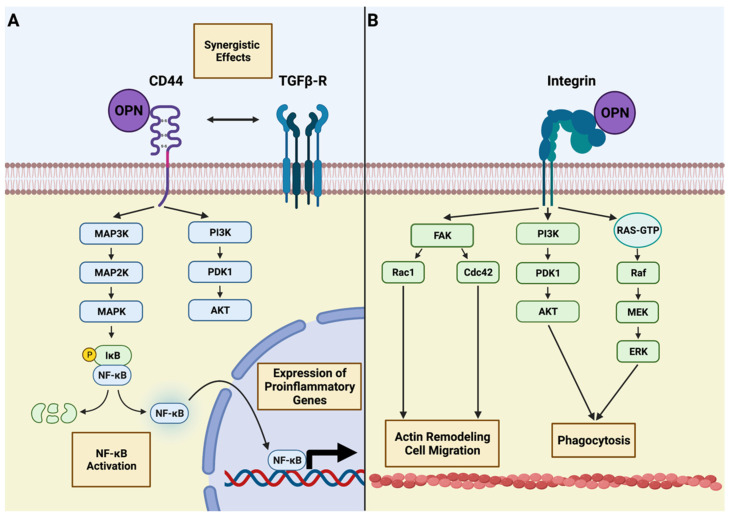
Osteopontin-related Pathways in Microglia. The interaction of OPN with CD44 leads to the engagement of several signaling pathways. (**A**) The activation of the phosphatidylinositol 3-kinase (PI3K)/AKT-pathway may be triggered by CD44 activation, followed by activation of the mitogen activated protein kinase (MAPK)-signaling pathway leading to the phosphorylation of the inhibitor of NF-κB (IκB) and the subsequent activation of the NF-κB pathway, which causes the expression of proinflammatory genes on a molecular level. Moreover, CD44 and TGFβ-R are known to interact with each other which may further promote the activation of proinflammatory signaling pathways. (**B**) The activation of integrin-related pathways leads to the activation of the focal adhesion kinase (FAK) with the subsequent recruitment of downstream molecules that ultimately promote actin remodeling and cell migration. The activation of PI3K, followed by 3-phosphoinositide-dependent protein kinase (PDK) 1 and AKT as well as the extracellular regulated protein kinases (ERK)-related pathway via Raf and Mitogen-activated kinase kinase (MEK) by integrins increases phagocytic activity in microglia.

Given the broad expression of OPN in various diseases of the CNS and the crucial role of microglia as its “housekeeper”, it is tempting to hypothesize that OPN and microglia may closely interact with each other. This notion is supported by the fact that several known receptors of OPN and its fragments are expressed by microglial cells (Figure 2).

One of these receptors is CD44 [51], which is expressed by several myeloid cell types, including microglia under neurodegenerative conditions such as AD [60]. CD44 is a transmembrane protein that is encoded by a 20-exon-containing gene [61]. Through alternative splicing of up to 12 of these exons and posttranslational modifications, CD44 variants (CD44v) may achieve an enormous heterogeneity representing the structural basis for its cell- and tissue-specific functions [62,63]. This pleomorphism is also reflected by its downstream signaling pathways. Although it is known that CD44 does not exert an intrinsic kinase activity, it may interact with several intracellular signaling molecules, including the Ras–mitogen activated protein kinase (MAPK) and phosphatidylinositol 3-kinase (PI3K)/AKT pathways (Figure 2A) [64]. One member of the MAPK family, p38 MAPK, was recently identified as a downstream target of OPN in retinal microglia in glaucoma [65]. Interestingly, it has been shown that several members of the MAPK signaling pathway, including the p38 MAPK, are upstream kinases of the NF-κB pathway [66,67], which is considered to play pivotal roles in inflammatory CNS disease, including MS [68]. Moreover, CD44 has been found to interact with transforming growth factor-β (TGF-β) receptors I and II [69]. TGF-β is a decisive cytokine for the maturation of microglia, which, in contrast to other CNS-associated macrophages [5], acquire their homeostatic gene expression signature in a TGF-β dependent manner [70] and whose proper function under homeostatic conditions is impaired in *Tgfbr2*-deficient mice [71]. Notably, the intracellular effects of TGF-β are mediated by the transforming growth factor-β-activated kinase 1 (TAK1) [72], which is expressed in microglia and whose activation leads to the subsequent phosphorylation of the inhibitor of NF-κB kinase (IKK) complex serving as the activating stimulus of NF-κB [73]. Previous studies have highlighted the role of microglia-derived TAK1 in CNS autoimmunity as the myeloid cell-specific deletion of the *Tak1* gene attenuates the disease severity in EAE mice [72]. Collectively, OPN increases the transcriptional activity of NF-κB in microglia directly through CD44–MAPK signaling and indirectly through CD44–TGFβR1/2-mediated TAK1 activation. The central role of the NF-κB pathway is further supported by the fact that microglial ablation of the NF-κB regulator protein A20 leads to an activated phenotype under homeostatic conditions and exacerbates the neuroinflammatory response of microglia after lipopolysaccharide (LPS) challenge [74]. Together, current data support the notion that CD44 and its associated downstream signaling molecules are present in microglia under diseased conditions, which have been previously identified as important modulators of microglial function. However, the exact extent by which MAPK signaling in microglia after OPN exposition is induced through the engagement of CD44 is poorly understood and was hampered by the lack of tools for microglia-specific gene targeting. Therefore, the recent advances in microglia-specific endonuclease mediated knock-in mouse models [75,76] such as the *Hexb^CreERT2^* mouse line [77] or binary Cre transgenic *Sall1^nCre^*:*Cx3cr1^cCre^* mice [78]. These mice have the advantage of maintaining their Cre-mediated recombination events leading to the excision and inactivation of genes of interest or the induction of fluorescent proteins but do not recombine in other macrophages, including monocytes in the peripheral blood [76]. Consequently, these models may pave the way for future studies investigating the OPN–CD44 axis in a more microglia-specific manner than previous lines such as the *Cx3cr1*^CreER^ model recombining in virtually all macrophages [76]. Further, the interaction between TGF-β and OPN-mediated signaling under healthy as well as in diseased conditions should be further clarified.

Other important mediators for OPN function are integrin receptors that bind to the N-terminal fragment of OPN after thrombin cleavage (Figure 2B) [49]. In general, integrins are heterodimeric proteins consisting of two subunits determining their ligand specificity and enabling their functional heterogeneity in different cell types and tissues [79]. In the case of microglia in the CNS, the main integrin receptor serving as a microglia marker is α_M_β_2_ integrin, which is also known as CD11b/CD18 integrin [80]. Moreover, microglia express several members of the β1 integrin family, including α_4_β_1_ and α_5_β_1_ integrins and the α_V_ integrin family [81]. Given its suitability as a microglia marker, the function of CD11b has been studied in healthy and disease conditions. Previous studies have highlighted the role of CD11b in complement-dependent synaptic pruning [82] and the orchestration of microglial clustering around blood vessels in EAE [83]. Hence, these findings underline the importance of integrins for a proper microglial function, but similarly to CD44 and other membrane-bound receptors, they rely on intracellular signaling pathways communicating signals from outside the cell. One important pathway for integrin receptor signaling that may be found in microglia is mediated by the focal adhesion kinase (FAK) [84]. Current data suggest that the activation of the FAK/Rac1/cell division control protein 42 (Cdc42)-GTPase pathways ameliorates the migration capacity of microglia in mouse models of AD [85]. Interestingly, a study elucidating the effect of an RGD- and SLAYGLR-containing OPN peptide on microglia found that these domains are necessary for an integrin-dependent enhancement of microglial phagocytic activity, which is mediated by FAK, PI3K/AKT and extracellular regulated protein kinases (ERK) signaling pathways [86]. This is in line with the finding that ERK signaling has recently been found to play a pivotal role in microglia activation during AD [87]. Hence, the binding of OPN to integrin receptors on microglia such as CD11b may engage several downstream mediators that have a crucial influence on the microglial phenotype and thereby contribute to the adaption of these to changes in their local microenvironment. In the past, several pathological conditions in the CNS were identified that may lead to changes in OPN expression and concomitant changes in microglial states. Therefore, we will further focus on these conditions and discuss our current knowledge about how the interaction between OPN and microglial cells influences their onset and development.

## 5. The Role of Osteopontin and Microglia in the Diseased CNS

**Figure 3:**
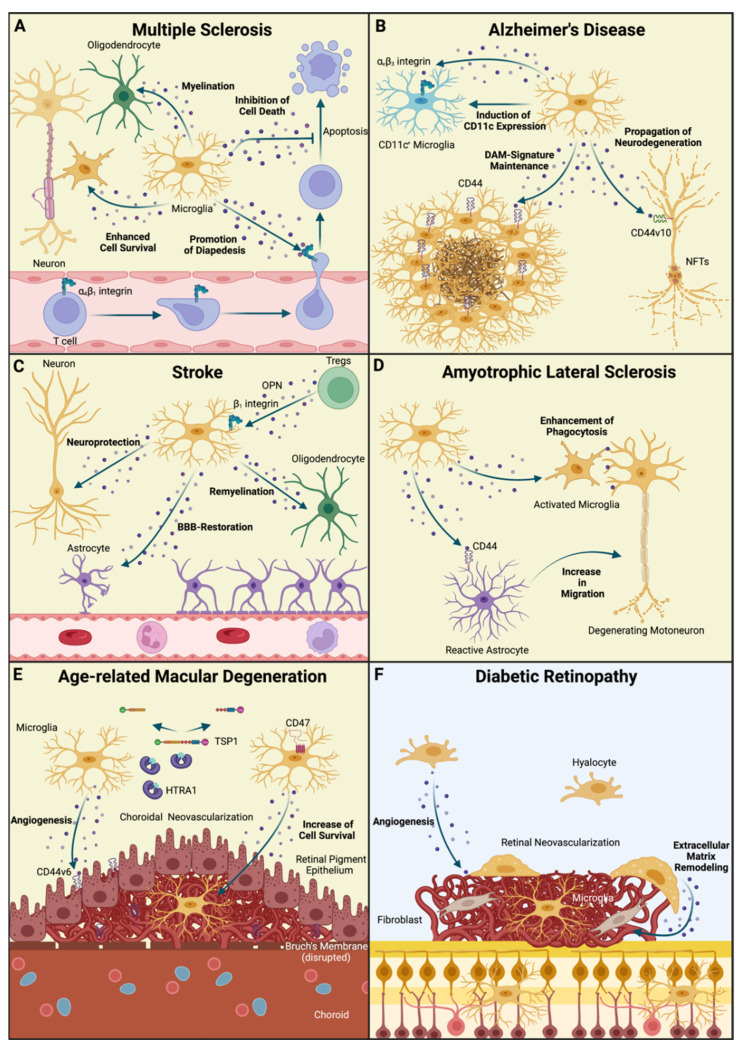
The Interplay between Microglia and OPN in CNS Pathology. (**A**) In MS, microglia-derived OPN (blue dots) promotes the influx of adaptive immune cells, including T cells whose survival is enhanced upon exposure to OPN (variants of OPN are indicated by gradual color differences and symbol size). Microglia increase their survival through OPN secretion in an autocrine and paracrine manner and stimulate myelination and oligodendrogenesis. (**B**) In AD, OPN is responsible for the acquisition and maintenance of the DAM signature and the CD11c-related phenotype of a microglia subset that is linked to amyloid plaques, here surrounded by disease-associated microglia (DAM). The OPN-dependent engagement of CD44v10 on neurons, enriched with neurofibrillary tangles (NFTs), may further propagate neuron cell death and hence contribute to neurodegeneration in AD. (**C**) In the context of stroke, the β1 integrin-mediated interaction between Tregs and microglia promotes remyelination and oligodendrogenesis. The restoration of the blood–brain barrier (BBB) is enhanced by OPN and confers neuroprotective effects. (**D**) During ALS development, microglia represent one of the main sources of OPN. OPN facilitates microglia-mediated phagocytosis through various mechanisms, including opsonization. In addition, OPN stimulates the migration of astrocytes from the white matter to the somata of degenerating motoneurons. (**E**) In AMD, macrophage-derived HTRA1 cleaves Thrombospondin (TSP-1), which in turn leads to increased secretion of OPN through reduced CD47-activation. OPN increases the survival of myeloid cells in the subretinal space and may exert proangiogenic effects through the engagement of CD44v6, supporting anastomosis between retinal and choroidal vasculature through the (disrupted) Bruch’s membrane. (**F**) In diabetic retinopathy, microglia are one of the main sources of OPN which may exert proangiogenic effects and influence fibroblasts in extracellular matrix remodeling. Notably, hyalocytes, the tissue- resident macrophages of the vitreous, may also contribute to the secretion of OPN during the development of retinal neovascularization.

Today, microglia and CNS-associated macrophages are widely regarded as key players in nearly all CNS pathologies [1,2]. Through recent developments in the field of single-cell analysis, including single-cell RNA-sequencing (scRNA-seq), Cytometry by Time-of Flight (CyTOF) or multi-omics approaches such as Cellular Indexing of Transcriptomes and Epitopes by sequencing (CITE-seq) [88], microglia cell clusters with distinct molecular characteristics have been defined across several diseases and disease models, including MS [28,42,89], AD [90], high-grade glioma [91,92,93], Huntington’s Disease [89], retinal degeneration [7] and AMD [8]. Despite the further need for a deeper functional characterization of microglial subclusters emerging under these conditions, we may draw the conclusion that yielding more information about the factors shaping these microglia phenotypes is mandatory for obtaining deeper insights into the disease’s pathobiology as well as developing myeloid cell-based therapies. Given the potential role of OPN as a therapeutic candidate for MS [19], we will discuss the role of OPN in these diseases with a particular emphasis on its relationship towards microglia (Figure 3).

## 6. Multiple Sclerosis

With approximately 2.5 million afflicted people around the world, MS represents an important source of disease burden in neurology [94]. MS is a neuroinflammatory disorder of the CNS that is characterized by the emergence of inflammatory lesions composed of B- and T-lymphocytes, monocytes and microglia leading to damaged myelin sheaths and demyelination [95] (Figure 3A). These inflammatory processes may lead to a plethora of neurological symptoms depending on the location of the lesions in the CNS, ranging from loss of sensation or motor function to vision loss or disorder of the bladder or the bowel [19,95]. From a clinical point of view, it is important to differentiate between relapse–remitting MS, where these neurological deficits have an acute onset followed by, in the majority of cases, a complete recovery after a period of time and progressive forms of MS that are characterized by a continuous worsening of the symptoms. Notably, the sterile neuroinflammation in relapse–remitting MS may lead to secondary axonal damage that is caused by the redistribution of neuronal ion channels such as transient receptor potential cation channel subfamily M member 4 (TRPM4) leading to ion disbalances and glutamate-mediated excitotoxicity [95,96]. Hence, these differences in pathophysiology should be kept in mind when discussing the role of myeloid cells including microglia and other important mediators.

Despite extensive research efforts in recent years, the exact role of microglia in the pathogenesis of MS remains elusive. Studies investigating the composition of human MS lesions have shown that microglia in active MS lesions exhibit a rather activated phenotype with an upregulation of activation markers such as human leukocyte antigen (HLA)-DR, which is nearly absent in microglia under homeostatic conditions [97], and the concomitant downregulation of homeostatic marker proteins, such as the purinergic receptor P2Y (P2RY12), transmembrane protein 119 (TMEM119) and the fraktaline receptor CX3C chemokine receptor 1 (CX_3_CR1) [98]. This is in line with previous studies investigating the transcriptional profile of murine microglia in EAE and human microglia derived from MS samples describing the emergence of microglial subclusters with downregulated expression of microglia signature genes such as the *purinergic receptor P2Y12* (*P2ry12*), *G-protein coupled receptor 34* (*Gpr34*), *Sialic acid binding Ig-like lectin H* (*Siglech*) and *Spalt-like transcription factor 1 (Sall1*) [28,42]. During the onset of the disease, microglia are considered to play an important role in the chemoattraction of encephalitogenic T cells. Therefore, they secrete an armada of proinflammatory cytokines such as C-X-C motif chemokine ligand 1 (CXCL1) or C-C chemokine ligand 2 (CCL2) which in turns increases the amount of infiltrating CD4^+^ T cells and monocytes [41,99]. Furthermore, microglia have been historically implicated in antigen presentation due to the increase in major histocompatibility complex (MHC) II molecule expression under neuroinflammatory conditions. However, recent studies have contradicted this point of view as microglia were found to be dispensable for T cell priming [42,100]. Moreover, microglia and other monocyte-derived macrophages contribute to grey matter pathology in later disease stages of MS by phagocytosing calcium-accumulating dendritic spines [101].

In addition to these disease-promoting roles, microglia display protective properties in MS [102]. Through TREM2-dependent phagocytosis, microglia can remove myelin and cell debris, which represents a necessity for subsequent remyelination. The importance of this pathway is underlined by the fact that TREM2-blockade in EAE exacerbates disease severity [103]. Furthermore, microglia depletion was shown to lead to increased inflammation, demyelination and axonal damage in a mouse model of secondary progressive MS with an overall increase in disease-related mortality [104]. Together, the role of microglia in MS and MS-related mouse models is far from being understood and future studies will be forced to gain deeper insights into the underlying spatiotemporal kinetics by which microglial function is determined in the course of the disease.

As already discussed in this review, OPN is significantly upregulated in MS lesions in comparison to healthy brain tissue [14] and has been found to be elevated in serum samples of MS patients [105]. Therefore, microglia and OPN have reciprocal effects on each other. Immunohistochemical studies in EAE mice indicate that OPN is expressed by several cell types in the diseased CNS, including endothelial cells, reactive astrocytes and, finally, microglia and infiltrating monocytes [14]. More recently, scRNA-seq analysis has provided evidence that OPN expression is restricted to certain microglia subpopulations in MS-affected brains and in a mouse model of cuprizone-induced toxic demyelination [28,106]. These data demonstrate that microglia are capable of secreting OPN, which in turn serves as a binding partner for several integrin receptors, including $\alpha_{4}\beta_{1}$ integrin, which is expressed on circulating T-lymphocytes and essential for T cell homing in MS [19] (Figure 3A). Notably, $\alpha_{4}\beta_{1}$ integrin is of great clinical significance as one of the most potent MS therapeutics, Natalizumab [107], exerts its function through the inhibition of this molecule [108]. Consequently, microglia derived OPN essentially contributes to the infiltration of peripheral immune cells in the inflamed CNS. Given the fact that the potency of Natalizumab is hampered by the possibility of an opportunistic infection with the John Cunningham (JC) virus causing progressive multifocal leukencephalopathy (PML), further targets for inhibiting leukocyte migration into the CNS are necessary. A recent study applying CyTOF to study myeloid cell populations across several CNS pathologies, including EAE, identified $\alpha_{5}\;\mathrm{integrin}\;(\mathrm{CD}49e)\;\mathrm{as}\;a\;\mathrm{potential}\;\mathrm{therapeutic}\;\mathrm{target}$ [89], which may also interact with OPN and other extracellular matrix proteins. Therefore, targeting OPN may still represent another promising target for MS therapy [19]. This notion is further supported by the identification of OPN-autoantibodies in a subgroup of MS patients that were correlated with a reduced number of relapses [109]. Beside the described role of microglia derived OPN in T cell homing, OPN itself has several effects on microglial cells. Similarly to T cells in EAE, whose survival is enhanced through OPN-mediated alterations in expression of proapoptotic proteins [59], OPN seems to increase microglial survival upon exposure [20]. As an adequate remyelination in sterile autoimmune inflammation highly depends on the resolution of microglial activation states, which has been shown to rely on necroptosis in a model of lysophospatidyl choline induced demyelination [110], a prolonged microglia survival may interfere with the resolution of the inflammatory state and hence hamper the remission. In addition, the expression of the intracellular OPN (iOPN) isoform in microglia, which may be induced by type I Interferon receptor activation, has been shown to be necessary for IL-17 producing T-helper cell response in EAE, whereas iOPN deficiency has been shown to delay EAE onset [111]. Notably, OPN may have opposing effects on oligodendrocytes as *in vitro* studies indicate that OPN enhances myelin basic protein synthesis and myelin sheath formation in OPC-like cells [112]. Despite these findings, current research suggests that the detrimental effects of OPN in EAE and MS outcompete its potential beneficial role and thereby provide a potential target for MS drug development.

## 7. Alzheimer’s Disease

AD represents the most common cause of dementia, thereby imposing enormous challenges on public health in aging societies [113]. The clinical presentation of patients suffering from AD is characterized by episodic memory loss and impairments in learning during the early stages of the disease, which is followed by heterogenous symptoms in later stages affecting executive functions, visuospatial functions and behavior [113,114]. In its sum, these symptoms may lead to severe life quality impairment for patients and caregivers. Whereas the clinical aspects of AD are largely known, the underlying pathobiology is still poorly understood. Neuropathological studies have revealed two prominent histopathological features in AD. On the one hand, the brain of AD patients is characterized by the accumulation of amyloid depositions in the extracellular space forming amyloid plaques [115]. The accumulation of amyloid can be found at an early stage of AD development and can even be detected in asymptomatic patients in the preclinical stage of disease [116]. To provide a mechanistic framework for AD pathophysiology, the “amyloid cascade hypothesis” has been proposed in which amyloid deposition is considered to be the initial pathophysiological event in AD giving rise to the subsequent pathological cascade [117]. However, clinical trials with drugs targeting the amyloid synthesis or deposition [118,119] have failed to ameliorate cognitive dysfunction, raising concerns as to whether the amyloid cascade theory adequately describes AD pathobiology [120]. As a second neuropathological hallmark, neurofibrillary tangles (NFT) composed of hyperphosphorylated tau protein can be found in the somatodendritic compartment of neurons from AD patients [115] (Figure 3B). NFTs seem to represent a driving force behind the neurodegenerative processes in AD as its amount is, in contrast to amyloid accumulation, strongly correlated with cognitive deficits [121]. Furthermore, the tau pathology seems to spread across neuroanatomically defined neural networks, which is the histopathological basis for the Braak staging of AD [122].

As a third hallmark of AD, recent years have highlighted the role of microglia activation in AD, although it is sometimes misleadingly subsumed as neuroinflammation despite the absence of typical hallmarks of inflammation. Histopathological studies indicate that microglia form clusters around amyloid plaques in mouse models of AD and in AD patients [123,124] (Figure 3B). Morphologically, these cells seem to exhibit a rather activated appearance with signs of cytoplasmic swelling and shortening of processes [123]. Of note, these results are conflicted by other studies demonstrating that microglial morphology is rather ramified in the surrounding of amyloid depositions but exhibits a dystrophic (senescent) phenotype with seemingly fragmented processes [125], that are connected through cytoplasm bridges as shown by electron microscopy [126], around sites of neurofibrillary degeneration. Nevertheless, it is commonly accepted that AD leads to severe alterations in microglial cells on a transcriptional level. Recent studies combining in-depth genotyping with transcriptional profiling of microglia in several CNS regions have provided evidence that several gene loci convey an increased AD risk through the alteration of the microglial transcriptome [127,128]. Furthermore, scRNA-seq has provided deeper insights into microglial states in 5xFAD transgenic mice, a mouse model for familial AD containing transgenes for human Amyloid Precursor Protein (APP) and Presenilin 1 (PSEN1) with 5 AD-linked mutations, and has revealed the emergence of DAMs with a unique gene expression profile characterized by the upregulation of genes associated with lipid metabolism and phagocytosis such as *Trem2, ApoE, Lpl* and *Cst7* [43] (Figure 3B). In the human setting, microglia in AD seem to display a higher grade of heterogeneity as the expression of these DAM signature genes can be found in several microglia clusters [90]. Interestingly, *APOE* and *TREM2* are known risk genes for familial AD [129]. Therefore, APOE is known to be associated with amyloid plaques in AD [130] and serves as a ligand for TREM2, which is known to be responsible for the acquisition of the DAM-phenotype [131]. On a functional level, APOE is responsible for orchestrating the microglial response against amyloid pathology as *Apoe*-deficient mice show a reduced amyloid compaction with a concomitant increase in dystrophic neurites [132]. TREM2-deficiency, on the other hand, leads to a wider spreading of tau pathology, which is associated with reduced microgliosis [133]. Together, microglia may exert protective roles in AD by limiting the spreading of amyloid- and tau-related pathologies and thereby decelerating neurodegeneration. However, microglia may also have a detrimental effect in AD. It is known that Aβ phagocytosis in microglia may lead to subsequent NLRP3 inflammasome activation whose genetic ablation in APP/PS1 mice ameliorates cognitive impairment [134]. Furthermore, Aβ oligomers may trigger complement factor 3 (C3)-dependent pruning of synapses leading to synapse loss in AD [135]. Finally, a recent study applying intracerebral Aβ grafting in rodents provided evidence that microglia phagocytosing Aβ may contribute to the Aβ dissemination into unaffected CNS regions [136]. In summary, the role of microglia in AD is not completely understood and is very likely to depend on disease-stage; therefore, further studies investigating the role of microglia in the human setting are urgently needed.

Similar to MS, OPN has raised the attention of scientists in AD research. Increased OPN expression has been observed in the brain [137] and the cerebrospinal fluid [138] of AD patients. One important source in the context of AD are microglia as the *Spp1* gene encoding OPN has been found to be an integral part of the DAM signature [43]. Notably, microglia concomitantly upregulate the expression of CD44, which enables OPN to form a positive feedback loop with its own receptor in an autocrine or paracrine manner [131]. Hence, OPN is largely involved in maintaining the DAM-signature in microglia during AD (Figure 3B). This notion is supported by the findings of a recent study in which OPN expression in the healthy CNS was largely restricted to a CD11c^+^ microglia subset that is maintained through the engagement of $\alpha_{V}\beta_{3}$ integrin [139]. In addition, OPN-deficient microglia have been found to lose CD11c-expression *in vitro* and in hippocampal organotypic slice cultures, which underlines the importance of OPN for the preservation of this CD11c-related phenotype [139]. As CD11c has been previously described as an integral part of the DAM signature [43] and CD11c^+^ microglia have been found to be in close proximity to amyloid plaques, the role of the OPN-$\alpha_{V}\beta_{3}$ integrin axis in CD11c^+^ microglia in AD should be addressed in future studies (Figure 3B).

Besides its effects on microglia, OPN may also influence surrounding cells in AD. It is known that CD44 and its splicing variants are differentially expressed across the cell types in the CNS with a prominent upregulation of CD44v6 and CD44v10 in neuronal cells in the brains of AD patients [140]. Interestingly, it has been shown that a CD44v10 inhibition through RNA-interference or monoclonal antibody treatment protected neurons from Aβ-mediated neurodegeneration [140]. Therefore, microglia-derived OPN may exert detrimental effects on surrounding cells by further propagating neuronal cell death and thereby accelerating neurodegenerative processes. Of note, OPN contributes to the senescence-associated secretory phenotype, which is characterized by the expression of several proinflammatory molecules and can be found in microglia during aging [141]. This is in line with a study describing an increase in *SPP1* expression with age in humans [91]. As the aged CNS is characterized by dystrophic microglia that can be regarded as the morphological correlate of cellular senescence processes [142], the role of OPN in dystrophic microglia may be of particular interest for the field. Furthermore, the differential role of OPN and its related pathways during physiological aging and neurodegeneration may be another promising path toward a deeper understanding of AD biology and the involvement of microglia in the underlying neurodegenerative process.

## 8. Cerebrovascular Disease

Due to its vital role for the body of mammals, the CNS requires a continuous and sufficient blood supply with adequate oxygen and nutrient support to fulfill its duty. A perfusion deficit, as it occurs in patients suffering from stroke, leads to severe and life-threatening CNS dysfunction. Due to the increasing prevalence of cardiovascular risk factors, such as hypertension, diabetes or hyperlipidemia [143], in western societies, stroke has become one of the leading causes of mortality and disability worldwide [144]. Despite significant improvements in preclinical and clinical treatments for acute stroke [145], the long-term processes following cerebral ischemia are still far from being understood and sufficiently addressed in the therapeutic setting. This may be partly explained by the fact that stroke is accompanied by a neuroinflammatory response involving both the innate and adaptive immune system. Cerebral ischemia initiates a series of molecular events altering the expression of adhesion molecules, including ICAM1 and VCAM1 in endothelial cells causing enhanced leukocyte adhesion and blood vessel occlusion [146]. Furthermore, the disruption of the blood–brain barrier (BBB) goes along with the influx of neutrophils and monocytes contributing to neuroinflammation in stroke by secreting proinflammatory cytokines and proteolytic enzymes such as MMPs [147]. This innate immune response is complemented by systemic changes initiated by the adaptive immune system. Cerebral ischemia and the associated death of neuronal cells leads to the release of previously cryptic CNS antigens into the systemic circulation where they encounter B and T lymphocytes in secondary lymphoid organs [148]. This provides a mechanistic framework for the discovery of autoreactive CD4^+^ and CD8^+^ T lymphocytes, as have been found in a mouse model of stroke, potentiating neuronal damage in stroke through autoimmune inflammation [149].

As the tissue-resident macrophages of the CNS, microglia are largely involved in the pathophysiology of cerebrovascular diseases, including stroke. Activated microglia exhibiting an amoeboid morphology can be found in the murine brain as early as 30 min after ischemia induction, thereby preceding neuronal cell death in the affected CNS region [150,151]. Even though *in vitro* studies have suggested proinflammatory properties of microglia in stroke with the potential to preferentially cause disadvantageous outcomes [151], studies in mice provide compelling evidence that the role of microglia in stroke is rather beneficial as microglia depletion worsens the outcome of stroke with a significant increase in infarct size [152]. On a functional level, microglia may protect neurons from excitotoxicity [152], reduce astrocyte-derived production of proinflammatory cytokines [153] and phagocytose infiltrating neutrophils to limit their tissue-damaging effects in stroke [154]. However, these neuroprotective functions should be treated with caution as they largely rely on studies in mouse models. Furthermore, patients with stroke exhibit a high grade of multimorbidity, which may augment microglial response to stroke in a rather detrimental way [102]. Exemplarily, a recent study by Jackson and colleagues has shown that microglia depletion in diabetic mice with experimentally induced stroke ameliorates cognitive impairment after cerebral ischemia [155]. Given the increasing spread of “civilization disorders” such as hypertension and diabetes, the function of microglia in stroke patients with these comorbidities will require further investigation.

It is well accepted that OPN is an important mediator in stroke pathophysiology (Figure 3B). OPN expression is upregulated in microglia surrounding the infarcted area as early as 24 h after experimental stroke induction and in microglia and infiltrating macrophages in the infarct area at 48 h after induction [156]. Mechanistically, OPN seems to exert neuroprotective functions in an RGD-domain and AKT/MAPK-signaling dependent manner in in vivo and *in vitro* models of ischemia [157]. This is supported by the findings of a study demonstrating that OPN-deficient mice exhibited an increased post-ischemic neurodegeneration in the thalamus and enhanced microglial activation [158]. Interestingly, this process seems to depend on inducible nitric oxide synthase (iNOS) as the administration of an iNOS inhibitor has been shown to lead to increased neuronal survival [158]. Furthermore, macrophage-derived OPN accumulates on the surface of dead neuronal cells, implicating a role for OPN in opsonization and macrophage-mediated phagocytosis [159]. In addition, OPN orchestrates the coverage of neovessels in the infarcted area by astrocytic feeds and thereby reduces the leakiness of the BBB [160]. Hence, OPN represents an important link between microglial cells and the proposed neuroprotective effects in cerebral ischemia.

In addition to these functions of microglia-derived OPN, it directly influences microglial phenotype and function. The exposure of microglia to OPN causes a shift in microglia phenotype from a rather proinflammatory to a regenerative, anti-inflammatory phenotype [161]. Notably, intranasal administration of OPN recapitulates these effects on microglial polarization and phagocytic activity through RGD-dependent mechanisms and thereby highlights the therapeutical potential of OPN [162]. Other important sources of OPN are regulatory T cells (Tregs), which infiltrate the diseased CNS during stroke and whose function is considered to be neuroprotective [163] (Figure 3C). A recent study by Shi and colleagues suggests a role for Treg-derived OPN in microglia-mediated white matter repair [164]. Here, the authors demonstrated that Treg-depletion worsened long-term recovery from stroke. On a mechanistic level, Treg-derived OPN binds to β1-integrin receptors on microglia which subsequently enhance oligodendrogenesis and remyelination [164] (Figure 3C). In summary, OPN and microglia exhibit an intimate relationship in stroke with rather beneficial functions for the clinical outcome. However, the role of OPN in stroke-related diseases as atherosclerosis and diabetes should be further disentangled as in this early phase of disease that may ultimately culminate in cerebrovascular dysfunction, OPN may exert opposing effects and should be therefore addressed differently.

## 9. Amyotrophic Lateral Sclerosis

There are an estimated 300,000 people suffering with Amyotrophic Lateral Sclerosis (ALS) in the world at any given time, which is predicted to significantly increase by 2040 [165]. Typically diagnosed during the fifth decade of life, ALS is a fatal neurodegenerative disease characterized by the degeneration of motoneurons in the brainstem and spinal cord and loss of descending motor tracts [166]. The first symptoms and the course of the disease are extremely variable. However, over time, all patients lose control of their muscles, which leads to loss of independence in their daily life and culminates in death. Despite intense research, the causes of this disease are still very poorly understood. Genome-wide association studies have identified variants in more than 30 genes that may convey an increased risk of developing ALS [166,167]. However, these genetic causes only account for roughly 10% of the patients. No environmental or behavioral causes have been found for the remaining sporadic ALS patients. Even though several aberrant physiological processes have been implicated in ALS pathogenesis, including excitotoxicity, oxidative damage, the formation of protein aggregates and mitochondrial dysfunction [168], ALS pathophysiology largely remains in the dark. A small fraction of cases termed familial ALS (fALS) are due to a variety of genetic mutations, with 20% of fALS cases due to dominantly inherited mutations in the *superoxide dismutase 1* (*SOD1*) gene [169]. SOD1 is a ubiquitously expressed, 32 kDa homodimeric cytosolic protein that is mainly responsible for the dismutation of free superoxide radicals into molecular oxygen [170]. In 1994, Gurney and colleagues [171] developed transgenic mice that overexpressed mutant SOD1 (mSOD1) and were characterized by progressive motoneuron degeneration, which recapitulated several pathophysiological hallmarks of ALS, including cytoplasmic mislocalization of TAR DNA-binding protein 43 (TDP-43) at the end-stage of the disease [172]. Of note, TDP-43 was found to play a role in the phagocytic capacity of microglia rendering them dysfunctional and hyperphagocytical with eventual detrimental effects to tissue homeostasis [173,174]. The majority of SOD1 mutants retain at least partially normal enzyme activity and ablation of the murine SOD1 gene does not culminate in motoneuron pathology [175], indicating that the pathogenic nature of mutated SOD1 (mSOD1) is through a toxic gain-of-function rather than a loss-of-function.

While motor neurons are the primary cells affected in ALS, other cell types, including microglia, have been shown to be involved in disease progression suggesting that neuroinflammation plays an important role in the pathogenesis of ALS disease. Significant levels of microgliosis, microglia proliferation and activation have been observed in the spinal cord of ALS patients at autopsy [176]. In addition, evaluation of ALS patients at various stages of their disease with positron emission tomography (PET) has shown microglial activation in the motor cortex, pons, dorsolateral prefrontal cortex and thalamus with a positive correlation between the extent of microgliosis and the severity of ALS [177]. In a mouse model of ALS, Don Cleveland’s group assigned a “non-cell autonomous” function to microglia in transgenic mSOD1 mice, the animal model of familial ALS. Selective inactivation of mutant *SOD1^G37R^* using a random integration to induce Cre expression under the control of 1.7 kb murine *Itgam* promotor fragment (*Itgam*-Cre) that yields excision of loxP-sites flanked genes only in the myeloid lineage, significantly prolonged the late stage of the disease [178]. In agreement, replacement of mSOD1-expressing microglial cells with wild-type microglia extended the survival in PU.1 knockout mice with mSOD1 and decreased motoneuron loss pointing toward a disease promoting role of microglia in this disease [179]. Recently, we performed high-dimensional analysis of cell surface markers and cytokines of microglia in the late stage of the disease in the mSOD1 model using CyTOF and identified three populations of microglia [89]. Moreover, we observed that all three populations expressed pro-inflammatory cytokines, such as TNFα, interleukin 6 (IL-6) and granulocyte-macrophage colony stimulating factor (GM–CSF) suggesting microglia could have a role in the pathogenesis of ALS due to their inflammatory nature [89]. However, ablation of 50% of reactive microglia in mSOD1 mice did not affect the rate of disease progression in mSOD1 mice [180]. These findings already implicate that healthy microglia may exert beneficial effects but removing microglia exhibiting an activated phenotype is not sufficient to suppress ALS progression, which underlines the importance of other factors for ALS pathobiology. Some of these factors are other myeloid cell populations that may be involved in ALS development. Given the fact that peripheral monocytes may represent another source of myeloid cells in ALS pathogenesis, we investigated the contribution of monocyte-derived macrophages to microgliosis observed in mSOD1 mice [181]. Using the parabiosis model, we demonstrated that despite the presence of microgliosis, there is no evidence of recruitment of blood-derived monocytes into the resident microglial pool [181]. Interestingly, recent data suggest that modifying peripheral macrophages, including nerve-associated macrophages, mitigates the microglia response in mSOD1 mice and even leads to increased survival [182]. Therefore, the crosstalk between myeloid cells in the peripheral nervous system and the CNS may represent a new promising avenue for developing myeloid cell-based therapies for ALS, which requires further investigation.

In the context of ALS, the expression of OPN has been investigated to understand whether it has a neurotoxic or neuroprotective role. The expression of OPN has been evaluated in CSF, neurons in the cortex and the spinal cord, microglia and astrocytes (Figure 3D). Two separate studies in ALS patients measured the level of OPN in the CSF of sporadic ALS patients and controls in the hope of finding a biomarker using ELISA method [183,184]. They reported an elevated level of OPN in the CSF of sporadic ALS patients compared to the controls [183,184]. In cortical neurons, two studies examined OPN with immunohistochemistry and demonstrated that the level of OPN was not significantly different between ALS patients and healthy control group [185,186]. However, one group demonstrated that OPN level was significantly reduced in the primary motor cortex and the spinal cord of ALS patients [186]. The same study reported that microglial OPN levels significantly increased in brain tissues from ALS patients compared to normal samples. In astrocytes, however, the expression level of OPN was not significantly different between ALS patients and the control group [186]. In animal model studies, transcriptional profiling revealed a significant upregulation of several neuroprotective and neurotoxic factors in microglia from mSOD1 mice including *Spp1* encoding OPN [187]. Concomitantly, CD44 is induced in microglia and astrocytes in mSOD1 mice, which may consequently serve as the mediator for OPN function in ALS [188] (Figure 3D). Indeed, the OPN-dependent engagement of CD44 in astrocytes from mSOD1 mice has been shown to increase the migration of these cells from the white matter into the grey matter harboring the motoneuron somata [189]. Moreover, OPN promoted microglia-dependent phagocytosis through opsonization [189]. Interestingly, the abundant expression of OPN can be found in different CNS regions in mSOD1 mice, whereas the expression of other proinflammatory molecules such as CCL2 has been shown to be largely restricted to the spinal cord [190]. These data point towards a “CNS-wide” role of OPN in ALS pathology. However, given the fact that OPN expression in ALS is not restricted to microglial cells, the relative contribution of microglia in comparison to other cell types, including neuronal cells, needs to be clarified in further studies. Furthermore, several independent studies have demonstrated that the expression of OPN mRNA significantly increased in spinal cord microglia during the disease progression [190,191]. While both human and animal studies suggest that the increase in OPN level in microglia during the progress of ALS might play role in ALS pathogenesis, more extensive studies are required to understand whether OPN expression in microglia plays a rather neurotoxic or neurotropic role at the different stages of ALS disease. This understanding may ultimately open a new chapter in the development of drugs for this fatal CNS disease.

## 10. Age-Related Macular Degeneration

In addition to the brain and the spinal cord, the retina is regarded as an integral part of the CNS as it is composed of neural tissue and develops from an evagination of the diencephalon [6]. Given its prominent role in visual perception, retinal disease may severely impair life quality. One of the major causes of severe vision loss in elderly people of western countries is age-related macular degeneration (AMD). Epidemiological studies estimate that until 2040, about 288 million people around the world will suffer from AMD, underscoring the importance of this disease for global health and the requirement for the development of adequate therapeutics [192,193]. In its early stages, AMD is characterized by the accumulation of extracellular deposits called “Drusen”, which are composed of lipid deposits, proteins and minerals [193]. In later stages, two different types of AMD may be distinguished. Whereas the “dry form” of AMD culminates in the geographic atrophy of the retinal pigment epithelium (RPE), photoreceptors and the innermost layer of the choroid, the choriocapillaris, the neovascular form of AMD, is accompanied by a disruption of Bruch’s membrane between the RPE and the choroid, the formation of pathological vessels called macular neovascularization (MNV) and the subsequent formation of subretinal fibrosis [194]. Despite the development of monoclonal antibodies targeting vascular endothelial growth factor (VEGF) in neovascular AMD [195], there is still an urgent need for further treatment options as patients may not respond to anti-VEGF treatment or develop fibrotic scars under anti-VEGF-therapy in long-term follow up [194,196,197].

Although excessive research efforts have been made in recent years, the underlying pathophysiology of AMD is still not properly understood. Our current knowledge about AMD includes genetic risk factors, environmental factors and immunological processes contributing to a multifactorial disease process [193,198]. Interestingly, several parts of the innate immune system have been linked to the underlying neuroinflammatory process in AMD [198]. Several genome-wide association studies have identified genetic variants in complement factors as risk factors for AMD [199,200], which is corroborated by studies demonstrating that complement inhibition attenuates the formation of choroidal neovascularization (CNV) in mouse models of AMD [201]. As the resident immune cells of the retina, retinal microglia are largely involved in AMD pathogenesis. Histopathological studies have demonstrated that microglia accumulate in the subretinal space (SRS) of AMD donor eyes and exhibit a rather amoeboid, activated morphology [202,203]. This histomorphological finding correlates with the bulk RNA-seq analysis of human CNV membranes in which macrophages were found to represent one of the predominating immune cell populations [204]. To gain deeper insights into the molecular heterogeneity exhibited by microglia in AMD as it was previously described for other pathological conditions of the CNS [205], scRNA-seq analysis was performed and revealed the emergence of DAM-subpopulations in a mouse model of laser-induced CNV [8,9], which represents a popular model for neovascular AMD [206]. These cells are characterized by the downregulation of homeostatic genes such as *Gpr34* and *Csf1r* and the concomitant upregulation of glycolytic enzymes, indicating a proinflammatory phenotype [8,207]. These data support the notion that microglia are crucial responders to the neuroinflammatory process in neovascular AMD. Furthermore, microglia seem to have disease-modulating effects on CNV formation as experiments in *Irf8*-deficient mice, which exhibit a rather immature microglial morphology and transcriptional profile, showed increased CNV-sizes after laser treatment [208]. Given the lack of treatment options for AMD, microglia-based therapeutic approaches are of great relevance for the field. Indeed, several molecules in microglia have been investigated for their therapeutic potential. Mechanistic studies have shown that Translocator Protein (TSPO) in retinal microglia is upregulated during inflammatory conditions and that its conditional depletion or pharmacological inhibition by synthetic ligands prevents microglia reactivity, neoangiogenesis and reactive oxygen species (ROS) production [209]. Furthermore, Interferon-β signaling seems to have beneficial effects on retinal microglia during AMD as systemic Interferon-β administration has been shown to significantly attenuate microglia response during the early phase of CNV, accompanied by decreased CNV sizes [210].

**Figure 4:**
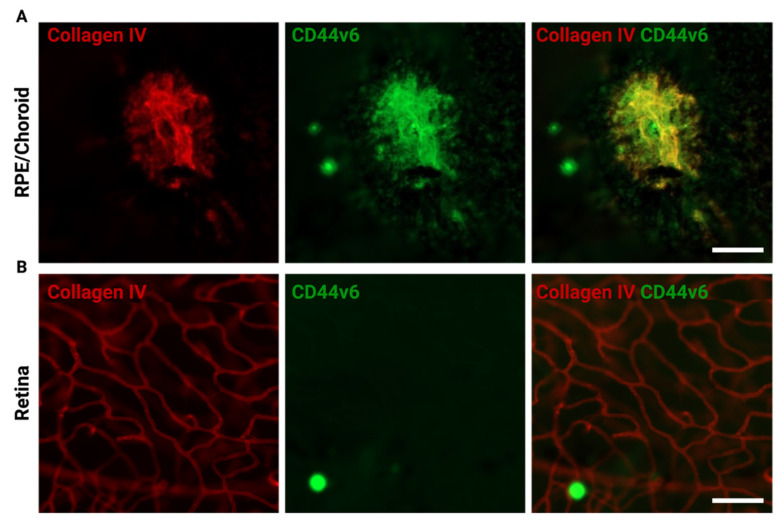
Expression Pattern of CD44v6 in Laser-induced Choroidal Neovascularization (CNV). (**A**) CD44v6-expression in proliferating endothelial cells in a mouse model of laser-induced CNV. (**B**) Absence of CD44v6 in quiescent retinal blood vessels. Scale bar represents 100 µm. Primary antibodies were monoclonal rat anti-CD44v6 (clone 94A) and polyclonal rabbit anti-Collagen IV, visualized by secondary antibodies chicken anti-rabbit Alexa Fluor^TM^ 647 and donkey anti-rabbit Alexa Fluor^TM^ 488. Whole-mount staining was performed as described previously [8].

Another molecule that has gained attention in AMD research is OPN (Figure 3E). Comparative transcriptome analysis has revealed that the *Spp1* gene encoding OPN is among the top upregulated genes in laser-induced CNV and CNV membranes from patients with neovascular AMD [17]. Furthermore, it has been shown that OPN is abundantly expressed by retinal microglia in CNV membranes and is involved in the process of myeloid leukocyte migration and tissue modeling [17]. On a molecular level, OPN may exert proangiogenic effects through the integrin receptor-dependent engagement of PI3K/AKT- and ERK1/2-related signaling pathways in endothelial cells [211]. Of note, we found that the CD44v6 variant is selectively expressed by blood vessels in RPE/choroid during CNV-formation, but not in retinal vasculature indicating that OPN may benefit from the selective expression of its receptor during CNV formation highlighting its potential as an anti-angiogenic treatment target (Figure 4). Interestingly, the intravitreal application of an OPN-blocking antibody significantly increased the laser-induced CNV-size, which contrasts with previous studies using a systemic anti-OPN antibody injection in CNV mice that reduced CNV lesion sizes significantly [212]. This phenomenon may be explained by several factors including the fact that systemic administration of anti-OPN antibodies may severely alter the balance of hematopoetic cells in the bone marrow with decreased numbers of monocyte–dendritic cell progenitors (MDPs) [213]. Of note, a recent study demonstrated that intravitreal injection of an OPN-blocking antibody reduced myeloid cell numbers and CNV sizes [16]. These results should be seen in light of different dosage and treatment regimens and may be thereby severely affected by kinetic-specific effects. Therefore, the context- and timepoint-specific role of OPN in CNV development needs to be clarified in further studies. Another important effect of OPN in CNV is its capacity to prolongate myeloid cell survival in the SRS [16], which is devoid of any immune cells under homeostatic conditions [202]. Mechanistically, it has been proposed that OPN represents the effector molecule of a pathway related to the serine protease High Temperature Requirement A1 (HTRA1). In this pathway, macrophage-derived HTRA1, which was shown to be one of the main hereditary risk factors for AMD [214,215], cleaves Thrombospondin which subsequently blocks CD47 activation and increases OPN release in subretinal myeloid cells [16] (Figure 3E). These effects on myeloid cells could be prevented by genetic deletion or pharmacological depletion of OPN underscoring its clinical potential [16]. Notably, we found that *HTRA1* is expressed at much higher levels in human retinal microglia in comparison to retinal microglia in mice [216]. Hence, the role of HTRA1 in microglial cells may be underscored in the murine setting and requires further investigation in humans.

Finally, aging is strongly associated with an increased expression of *Spp1* in retinal microglia and increased CNV-sizes in old mice in comparison to younger mice [217]. This finding recapitulates scRNA-seq analysis of human brain microglia showing a similar age-dependent increase in OPN expression [91]. Therefore, a deeper understanding of the immunosenescence processes in the myeloid compartment of the retina may pave the way for an improved understanding and new treatment options of age-related diseases such as AMD.

## 11. Diabetic Retinopathy

One of the main microvascular complications of diabetes is diabetic retinopathy (DR), which is considered to represent the main cause of blindness in the working population worldwide [218]. Whereas about 35% of patients suffering from diabetes develop early stages of DR called non-proliferative diabetic retinopathy (NPDR), about 7% progress to the development of proliferative DR (PDR), characterized by the presence of retinal neovascularizations (RNV) in the vitreous cavity [219,220]. Additionally, almost 7% of DR patients may independently develop diabetic macular edema (DME), which describes a particular form of NPDR with vascular leakage in the macula [220]. Although DR development is closely related to diabetes-associated risk factors such as diabetes duration and hyperglycemia, several other risk factors such as hypertension, obesity and anemia have been identified [221,222]. Furthermore, recent years have witnessed an enormous gain in information concerning the pathophysiological events underlying DR development. In the early stages without visible vascular alterations, the function of the neurovascular unit linking neuronal activity and the resulting oxygen demand to retinal blood supply is impaired [220]. In addition, this phase is associated with the extracellular accumulation of glutamate promoting excitotoxicity and the reduced expression of neuroprotective factors in the retina such as pigment epithelial derived factor (PEDF) [223]. In sum, these changes promote neuronal cell death in the retina. Moreover, proangiogenic factors such as VEGF and proinflammatory cytokines are upregulated in the diabetic retina causing leukocyte adhesion, endothelial cell death and blood–retina-barrier impairment [220,224,225,226]. In PDR, the excess in proangiogenic factors due to local hypoxia causes the recruitment of endothelial cells and the formation of RNVs whose blood vessels show a tendency towards bleeding and fibrosis [227] (Figure 3F).

Despite its microangiopathic character, DR exhibits a strong inflammatory component in which microglia play an important role. Myeloid cells, such as microglia, accumulate at sites of ischemia and retinal neovascularization in humans and in experimental models of retinal neovascularization [228,229,230] (Figure 3F). Furthermore, immunohistochemical studies in human eyes from DR patients identified microgliosis as a histological hallmark of DR progression [231]. This is corroborated by scRNA-seq analysis in akimba mice, a popular model for DR in which mice contain a mutation in the insulin 2 gene (*Ins2*) and overexpress VEGF [232], demonstrating a strong upregulation of signaling pathways related to immune system processes like antigen processing and presentation [233]. In DME, microglia have even been found to migrate to the subretinal space [231]. Mechanistically, microglia can secrete several proinflammatory cytokines such as tumor necrosis factor (TNF) α or CCL2 under hyperglycemic conditions driving the pathological changes in DR [234,235]. It is known that in streptozotocin-induced hyperglycemia, microglia-derived TNFα activates the NF-κB pathway in RPE cells and thereby reduces the expression of tight junction proteins, which is accompanied by an impairment of the outer blood–retina barrier between retina and choroid [236]. Further, microglia contribute to retinal vasomotor regulation through an CX_3_CR1-Angiotensinogen-dependent pathway, which has been shown to be dysregulated in the early phase of hyperglycemia [237]. This may be of particular interest for the translation into the clinical setting as studies taking advantage of optic coherence tomography (OCT) indicate that the early phase of DR is characterized by the accumulation of hyperreflective spots in the inner retinal layers, which are supposed to be aggregated microglia [238,239]. Preclinical evidence suggests that microglia proliferate in ischemic areas and RNV in a mouse model of oxygen-induced retinopathy (OIR) [229]. Systemic depletion of myeloid cells by liposomal clodronate results in a reduction in RNV in the OIR mouse model, suggesting a pro-angiogenic effect of myeloid cells [240]. In contrast, other studies show that intravitreally applied myeloid cells reduce the development of retinal neovascularization, indicating an anti-angiogenic effect of myeloid cells [241]. These seemingly contradictory results could be explained by a different function of resident microglia versus blood-derived infiltrating monocytes. Recent evidence further suggests that microglia acquire a glycolytic phenotype in the OIR model that leads to increased acetyl coenzyme A production and histone modifications promoting retinal neoangiogenesis [242]. Collectively, current literature indicates that microglia are key players in all stages of NPDR and PDR and thereby represent promising targets for DR therapy.

Due to the involvement of inflammatory processes in DR pathogenesis, several proinflammatory molecules, including OPN have been investigated in the past. Protein analyses of vitreous samples from DR patients confirms increased OPN levels [243]. Interestingly, OPN levels in the plasma seem to correlate with the clinical severity of DR [244] underlining the potential of OPN as a biomarker. On a molecular level, OPN increases the thickness of vascular basement membranes in RNV by upregulating the expression of Collagen IV [245]. This process seems to be mediated by the repression of microRNA-29 (miRNA-29). Interestingly, NF-κB activation has been shown to repress miRNA-29 [246]. Given the fact that microglia are the predominant immune cell population in mouse models of DR, including OIR [229], it is tempting to speculate that these cells represent a main source of OPN in DR (Figure 3F). In this context, retinal microglia were also identified as the main myeloid cell population in a murine model of retinal angiomatous proliferation (RAP) with low angiogenic potential but an increase in *Opn* expression, investigated by RNA-Seq [247]. In the human situation, a very recent study utilizing scRNA-seq to characterize the cellular landscape of fibrovascular membranes in humans identified microglia as the main source of OPN [248]. Interestingly, OPN was found to be the main mediator in microglia-fibroblast crosstalk, emphasizing its important role in tissue remodeling and fibrosis [248]. However, another myeloid cell population known as hyalocytes, the resident immune cells of the vitreous cavity, should be kept in mind when elucidating the role of the myeloid compartment during DR. Hyalocytes are myeloid cells exhibiting a distinct gene expression profile in comparison to microglia [249,250,251]. One of the most dominant transcripts in human hyalocytes under physiological conditions is *SPP1* encoding OPN [249]. Further, hyalocytes can be found in human RNV [230], which complicates the investigation of the microglia-specific contribution in OPN-production (Figure 3F). Notably, single-cell proteomics analysis using Imaging Mass Cytometry (IMC) indicates that a subset of HLA–DR expressing macrophages exhibits phenotypic characteristics of myofibroblasts as shown by α-smooth muscle actin (SMA) immunoreactivity [230]. Cell culture experiments with human hyalocytes have revealed that hyalocytes have the capacity to transdifferentiate into myofibroblasts [230]. In DR, myofibroblasts are known to express OPN [252] and thereby, hyalocytes have the potential to contribute to OPN-production through different pathways, which should be addressed in further studies.

## 12. Concluding Remarks

In recent years, the rapidly developing field of microglia biology has witnessed an immense number of discoveries that have changed previous dogmata and established a more sophisticated view of their role in the CNS. These discoveries have also influenced the role of important effector molecules, including OPN, as it became clearer that microglial heterogeneity and disease-specific microglial functions need to be considered when investigating the role of OPN during health and CNS pathology (Figure 3). The main purpose of this review was to highlight the role of microglia, OPN and its related signaling pathways in the CNS as well as to discuss their interplay across pathologies affecting the brain and the retina. It should become clear that as heterogenous as the CNS during health and disease might be, as diverse are the roles of OPN. However, current genetic tools may help us to interrogate open questions in this field that may represent the starting point for upcoming investigations. First, neuroinflammatory conditions such as MS in the brain or AMD in the retina cause severe alterations in the blood–brain- or blood–retina barrier, respectively, leading to a significant influx of peripheral immune cells with the capacity to secrete OPN. Furthermore, OPN may also be secreted by other cell types, including neurons and astrocytes, which additionally complicates the investigation of microglia specific OPN effects and functions in vivo. The usage of microglia-specific mouse lines [75,76] may help us to study the interplay between microglia and OPN in a cell type-specific manner. Another important factor which should be considered when discussing the role of microglia derived OPN is the temporal dynamic of the disease, which is accompanied by changes in the role of microglial cells at different disease stages. Whereas microglia in early stages may display beneficial properties, late stages may be associated with microglia dysfunction and worsened disease outcomes. Hence, the effects of OPN may similarly depend on the timepoint in which its role is investigated. Therefore, incorporating temporal information about gene or protein expression into experimental setup, as recently demonstrated for RNA timestamps [253], may lead us to new discoveries concerning microglia and OPN during health and disease. Finally, there is an urgent need for studies investigating the role of OPN in the human setting. Although the contribution of mouse models to the extensive knowledge gain in microglia biology cannot be negated, the validation of OPN-ascribed affects in humans [17] or advanced pluripotent stem cell-derived models is a prerequisite for further translation into the clinical setting.

In conclusion, the picture of the reciprocity between OPN and microglia become clearer as new technologies have enabled us to ask questions which were previously not addressable. However, the more we know, the more open questions remain with significant implications for patients suffering from CNS disease. Answering these questions will not only lift the fog of uncertainty concerning the molecular mechanisms on which this interplay is based, but also help us to establish the role of OPN as it is already implicated by its name, forming a bridge between fundamental questions in neuroscience and immunology and the development of new microglia-based drugs for patients suffering from still untreatable CNS disease [44].

## Data Availability

Not applicable.
